# The Future of Energy Storage: Advancements and Roadmaps for Lithium-Ion Batteries

**DOI:** 10.3390/ijms24087457

**Published:** 2023-04-18

**Authors:** Muhammad Adnan

**Affiliations:** Graduate School of Energy Science and Technology, Chungnam National University, Daejeon 34134, Republic of Korea; adnan@cnu.ac.kr

Li-ion batteries (LIBs) have advantages such as high energy and power density, making them suitable for a wide range of applications in recent decades, such as electric vehicles, large-scale energy storage, and power grids. However, in order to comply with the need for a more environmentally friendly society, the rapid development of LIBs with lower costs, increasingly higher energy and power density, and improved safety during charging and discharging is expected. Currently, the most popular type of rechargeable battery is the lithium-ion, which currently powers a range of devices from smartphones to electric cars. LIBs are superior to other battery systems because of their longer lifetimes, higher energy densities, and faster recharge times.

The key advantages of LIBs are their ability to produce high energy density, which allows them to store more energy in a smaller package and makes them ideally compatible for use in portable electronic devices such as laptops, smartphones, and tablets. Moreover, LIBs are also superior due to their longer lifetimes in comparison to other rechargeable batteries due to the use of more stable chemistry, which makes them less prone to degradation over time. Additionally, LIBs have fast recharge times, which is highly desirable for use in electric vehicles, where a fast recharge time can mean the difference between reaching to your destination or being stranded on the road. However, there are some concerns and drawbacks which need to be addressed. Among them, the key concerns regard their safety. If LIBs are not designed, optimized, and manufactured properly, they could be prone to overheating, which may lead to lethal fires and/or explosions. This is a very high concern for the motor vehicle aviation industry, in which LIBs have been directly linked with numerous high-profile incidents. The second biggest concern regarding LIBs is their environmental impact. LIBs still rely on the extraction of minerals such as lithium and cobalt; these extraction processes could have noteworthy impacts on local ecosystems. Moreover, the disposal of LIBs could also be challenging, because they contain toxic elements which can directly harm the environment and its surroundings.

Meanwhile, the growth of LIBs has had tremendous benefits for society, from longer-lasting smartphones to cleaner electric vehicles. Nevertheless, we must also be aware of the potential risks and environmental impacts that are directly associated with this astonishing technology. Because we are continuing to rely on LIBs to power our electronic devices and electric vehicles, it is highly important that we continue to address these concerns and make sure that this tremendous technology is being used in a safe and sustainable way. Therefore, with all of these aspects in mind, I, the Guest Editor of the Special Issue entitled “Material Design and Mechanisms of Lithium-Ion Batteries”, would like to thank the authors for their contributions in this Special Issue. I believe that these published outcomes will be helpful for researchers and scientists working in this field.

